# The effect of different test protocols and walking distances on gait speed in older persons

**DOI:** 10.1007/s40520-020-01703-z

**Published:** 2020-09-15

**Authors:** Sebastian Krumpoch, Ulrich Lindemann, Anja Rappl, Clemens Becker, Cornel C. Sieber, Ellen Freiberger

**Affiliations:** 1 Institute for Biomedicine of Aging, Friedrich-Alexander-University of Erlangen-Nürnberg, Kobergerstr. 60, 90408 Nuremberg, Germany; 2 Department of Geriatrics and Clinic for Geriatric Rehabilitation, Robert-Bosch-Hospital, Auerbachstr. 110, 70376 Stuttgart, Germany; 3 Department of Medical Informatics, Biometry and Epidemiology, Friedrich-Alexander-University of Erlangen-Nürnberg, Waldstr. 6, 91054 Erlangen, Germany; 4 Department of Medicine, Kantonsspital Winterthur, Brauerstr. 15, 8400 Winterthur, Switzerland

**Keywords:** Acceleration phase, Deceleration phase, Gait speed, Aged, Test protocol

## Abstract

**Background and aims:**

Walking is the core physical activity of older persons. The assessment of walking capacity is increasingly important for clinical purposes and clinical research. Differences between assessment tools and protocols for short walks to obtain gait characteristics can be responsible for changes, e.g., in gait speed from 0.1 to 0.2 m/s. The purpose of this study was to generate further knowledge for the harmonization and/or standardization of short walk-test protocols for assessing gait characteristics under supervised conditions.

**Methods:**

For this cross-sectional study, 150 community-dwelling older adults (mean age 80.5 ± 4.5 years) were recruited. Participants performed eight walks differing in the distance (8-versus 4-m), static versus dynamic trials and comparing different test speed instructions (usual versus maximal) on an electronic walkway.

**Results:**

A meaningful significant difference in mean usual gait speed was documented comparing the 4-m dynamic and static test protocol (0.12 m/s; *p* = 0.001). For the same comparison over an 8-m distance (dynamic versus static) and for the comparison between usual gait speed over 4-and 8-m, the differences in gait speed were smaller, but still statistically significant (*p* = 0.001).

**Conclusions:**

Gait speed was faster, if the test protocol did not include a static start or stop. The differences were greater for a shorter walking distance. This aspect should be considered for the comparison of study results and is particularly relevant for systematic reviews and meta-analyses.

## Introduction

Walking is a core activity of older persons and a relevant component to overall mobility. Evidence exists that a decline in gait speed of older persons is associated with many negative health outcomes, such as death [1], frailty [2] or hospital admission [3]. Gait speed is increasingly acknowledged as a “vital sign” [4]. To be used in clinical practice, gait speed measurements should be standardized and for clinical research the results should be at least harmonized [5].

Assessing gait speed is not trivial. Gait characteristics can be measured supervised in a research laboratory or unsupervised during daily activities by body-worn sensors [6]. It is increasingly recognized that supervised gait speed in controlled environments differs significantly from real-life non-supervised gait speed measured in the general older community [7] and according to disease status [8–10]. Monitoring gait speed in real life is not yet established. This requires assessments by body-worn sensors over several days [6]. Gait speed is measured predominantly in the gait laboratory or similar clinical settings. Instruments like electronic walkways or photoelectric barriers are established gold standards. They eliminate reaction time errors, which can occur when measuring gait speed manually.

Provided the test methodology is fully reported at all [5], research groups often use walking protocols with different gait conditions to test their study participants [7, 8]. This includes walking distances of 4-m to 10-m. Most protocols include a normal/usual/habitual walking condition and a fast walking condition without or with additional cognitive tasks. Additionally, standardized measurement of steady-state gait via short tests is problematic, because the real-life walking includes acceleration and deceleration affecting mean gait speed [11, 12]. For clinical routines, this is impractical as time and resources are limited. Therefore, clinical routines have simplified the gait assessment using only one condition. This plethora of approaches result in a difficult position for clinicians leading to different protocols since the landmark JAMA study by Studenski [1]. Other studies show that differences between assessment tools and protocols can be responsible for changes in supervised gait speed in a range of 0.1–0.2 m/s [13–15]. These are speed ranges that are considered as clinically meaningful [16].

The ongoing debate is now being influenced by regulatory agencies such as the European Medicines Agency (EMA) and US Food and Drug Administration. The EMA in a recent decision has adopted the Short Physical Performance Battery (SPPB) [17] as their current gold standard to assess mobility. This includes a 4-m supervised gait assessment from a standing start. It can be expected that this will establish a norm at least for clinical routines and additional testing will use this as a frame to discuss their findings.

The purpose of this study was to generate further knowledge for the harmonization (standardization) of supervised short walk-test protocols as well as data generalization. Additional methodological findings could increase and specify the significance of gait speed measurements, reduce the risk of errors in group- and sample comparisons and facilitate the constitution of standardized protocols in the scientific community [5]. It was hypothesized that usual gait speed is faster, if the test protocol does not include a static start or stop at the end. Furthermore, this study also examined whether this effect could be reproduced with maximal gait speed and if a difference between protocols would be greater for a shorter walking distance.

## Methods

### Subjects and design

For this cross-sectional study, community-dwelling older adults were recruited between May and December 2019 from an existing data pool and by flyers distributed in the greater area of a south-east German city. Participants had to be at least 70 years old, able to walk without a wheeled walker for 10-m and to understand and follow instructions. Exclusion criteria were orthopedic and/or neurologic problems, which caused walking problems (self-report). The study protocol was approved by the ethical committee of FAU Ethical Committee (43_19B) and all participants had to give written informed consent.

### Primary outcome measures

**Fig. 1:**
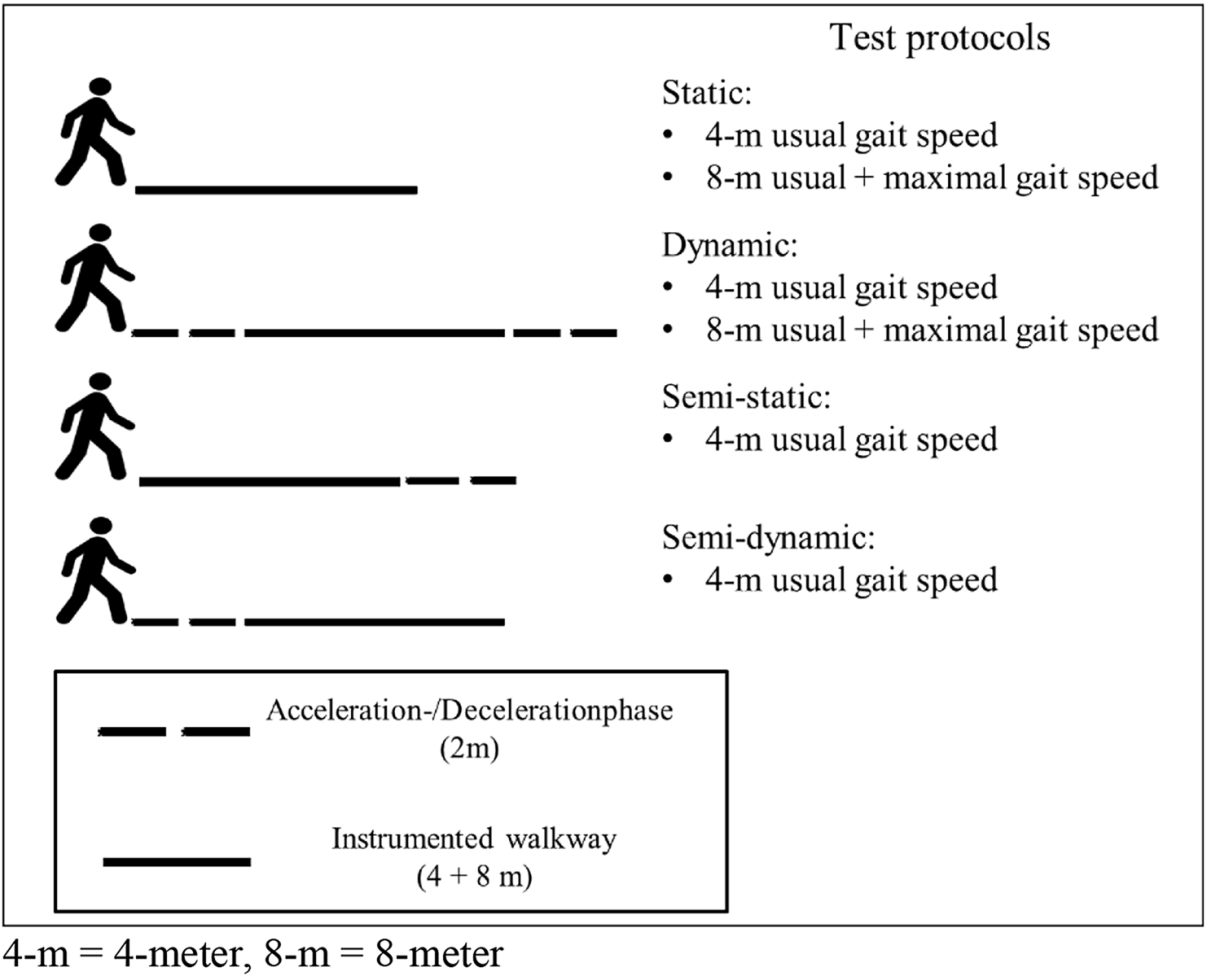
Description of the test conditions with regard to acceleration and deceleration. 4-m , 4-meter; 8-m, 8-meter

Gait analysis was performed on an instrumented 10-m-long walkway with embedded pressure sensors (Gold walkway, 972 cm long, active electronic surface area 792 × 610 cm, total 29,952 pressure sensors, scanning frequency 60 Hz, GAITRite, CIR Systems Inc., Franklin, USA) in a well-lit hallway. The validity and reliability of the GAITRite system has been shown in previous studies [18, 19]. The GAITRite system calculated gait speed as the ratio of the parameters Distance Travelled and Ambulation Time. Distance Travelled was measured on the horizontal axis from the heel center of the first footprint to the heel center of the last footprint, while Ambulation Time was recorded as the time elapsed between first contact of the first and the last footfalls. The protocol consisted of eight walks differing with respect to distance (7.9-versus 4.3-m; in the following indicated as 8-and 4-m, respectively), start/end protocols (static versus dynamic) and speed (usual versus maximal) (Table 1). The 8-m-long active sensor area of the instrumented walkway can be modified using the GAITRite software. The 4-m measurements were realized by computer-based reduction of the not required carpet length. Cones were placed on the side of the walkway to visualize the start and finish area for each test condition. The rationale for comparing a 4-m distance with an 8-m distance was the widely used 4-m distance as part of the SPPB [17] and another short, but somewhat longer distance which is also described in the literature [15]. Static walks involved exclusively the active carpet area. Dynamic and semi-dynamic walks included acceleration and deceleration phases of 2-m, which were partly outside the active carpet area (Fig. 1). The instruction for a static walk at usual pace was: “Please walk in your usual gait speed and come to a sudden stop when you have reached the cone.” The instruction for a dynamic walk at maximum pace was: “Please walk as quickly and safe as possible without running until you reach the indicated cone.” Participants were allowed to rest between walks as needed. The walks were carried out in randomized order.

**Table 1:** Test conditions of all walks with regard to gait speed, start/end and distance

Gait speed	Start condition	End condition	Distance (m)
Usual gait speed	Static	Static	4
Usual gait speed	Dynamic	Dynamic	4
Usual gait speed	Static	Dynamic	4
Usual gait speed	Dynamic	Static	4
Usual gait speed	Static	Static	8
Usual gait speed	Dynamic	Dynamic	8
Maximal gait speed	Static	Static	8
Maximal gait speed	Dynamic	Dynamic	8

### Descriptive measures/covariates

Demographic and anthropometric data, such as age, sex, body weight and height were recorded. Functional comorbidity [20] was obtained by questionnaires in a standardized interview. Cognitive function was assessed with the Montreal Cognitive Assessment [21]. The SPPB sum score [17] was used to describe the global physical function of the participants.

### Data analysis

Paired *t* tests analyzed the differences with regard to the inclusion or exclusion of the acceleration and deceleration on the 8-m walks in usual, as well as in maximal gait speed.

A one-way repeated measure analysis of variance (ANOVA) was used to examine the effect of four different combinations of acceleration and deceleration on gait speed over 4-m.

The effect of the walking distances (4-and 8-m), along with the effects of including or excluding the acceleration and deceleration and their interaction was analyzed by a two-way repeated-measure ANOVA. For all calculations, a significance level at *α *=* 0*.05 was used. Post hoc paired *t* tests with Bonferroni correction were applied. All analyses were performed using SPSS^®^ version 24.0 (SPSS, Inc., Chicago, IL, USA).

## Results

The data of 150 participants with a mean age of 80.5 ± 4.5 years (61% women) were included in the analysis. The description of the cohort in detail is presented in Table 2.

**Table 2:** Description of all (*n* = 150) participating older adults

Characteristic	Mean ± SD (range) or *n* (%)
Sex, female/male	92 (61)/58 (39)
Age [years]	80.5 ± 4.5 (71–93)
Body height [cm]	163.8 ± 10.5 (142–198)
Body weight [kg]	74.9 ± 16.2 (43.9–121.9)
Education [years]	13.7 ± 3.3 (8–28)
Body mass index [kg/m^2^]	27.8 ± 4.8 (17.6–43.3)
Short Physical Performance Battery (0–12)	10.9 ± 1.7 (4–12)
Montreal Cognitive Assessment (0–30)	25.4 ± 2.9 (13–30)
Functional Comorbidity Index (0–18)	3.6 ± 2.2 (0–9)

*SD* standard deviation; Note: better score values are underlined

A clinically meaningful difference in mean usual gait speed was documented for the comparisons between the 4-m dynamic test protocol and 4-m static test protocol (0.12 m/s) and between the 4-m dynamic test protocol and 8-m static test protocol (0.1 m/s). All mean gait speeds at different test conditions are presented in Table 3.

**Table 3:** Mean gait speeds of different test conditions of all participating older adults (n = 150)

Distance/speed	Test protocol Start	Test protocol End	Mean ± SD (range) [m/s]
4-m usual gait speed	Dynamic	Dynamic	1.23 ± 0.28 (0.25–2.01)
4-m usual gait speed	Static	Static	1.11 ± 0.26 (0.24–1.92)
4-m usual gait speed	Static	Dynamic	1.17 ± 0.26 (0.26–1.75)
4-m usual gait speed	Dynamic	Static	1.19 ± 0.28 (0.27–1.94)
8-m usual gait speed	Dynamic	Dynamic	1.20 ± 0.26 (0.33–1.80)
8-m usual gait speed	Static	Static	1.13 ± 0.27 (0.29–1.83)
8-m maximal gait speed	Dynamic	Dynamic	1.52 ± 0.35 (0.34–2.39)
8-m maximal gait speed	Static	Static	1.47 ± 0.34 (0.31–2.31)

*SD* standard deviation; 4-m = 4-meter, 8-m =8-meter

Differences between dynamic and static test protocols for both usual (*t* (*df* = 149) = 6.96, *p *= 0.001) and maximal (*t* (*df* = 149) = 4.85, *p *= 0.001) gait speed over 8-m were statistically highly significant.

Comparing the 4-m walks concerning four different test protocols, a repeated measures ANOVA with a Huynh–Feldt correction revealed that mean performance levels showed a statistically significant difference between measurements (*F* (2.83, 420.86) = 92.67, *p* < 0.001, partial *η*^2^ = 0.38). Bonferroni-adjusted post hoc analysis presented significant differences between all 4-m walks (Table 4).

**Table 4:** Levels of statistical significance (*p* values) of differences between test conditions for 4-m walks at usual gait speed analyzed by one-way repeated measures analysis of variance

	D/D	S/S	D/S	S/D
D/D		0.001**	0.001**	0.001**
S/S	0.001**		0.001**	0.001**
D/S	0.001**	0.001**		0.016*
S/D	0.001**	0.001**	0.016*	

*S* static; *D* dynamic

*Statistically significant difference in mean walking speed among four conditions with different test protocols; *p *< 0.05*, *p *< 0.001**

The two-way repeated measures ANOVA considering distance, test protocol (dynamic and static) and the interaction thereof displayed a statistically significant interaction between the effects of walking distance (4-m vs. 8-m) and test protocol (dynamic vs. static). Simple main effect analysis showed that test protocols had a significant impact on gait speed, but there was no overall distance-related difference between 4-m and 8-m walking speed (Table 5). Only when combined, there was a significant effect (interaction A × B).

**Table 5:** Two-way repeated measures analysis of variance on: main effects A and B, interaction effect A × B

Effect	*F*	*df*	Partial *η*^2^	*p*
Distance (A)	0.32	1	0.002	0.572
Test protocol (B)	194.27	1	0.566	0.001*
A × B	17.86	1	0.107	0.001*

*A* main effect of 4-m or 8-m walking distance; *B* main effect of acceleration/deceleration inclusion

*p < 0.001

## Discussion

As expected, we observed in our study a significant effect of the inclusion or exclusion of acceleration and deceleration phases on gait speed measurements over 4-and 8-m. This effect confirmed our hypothesis and was strongest, i.e., clinically meaningful and statistically significant, between 4-m walks with uniform test protocols in usual gait speed. Statistically significant differences were attained for 4-m walks with mixed test protocols in usual gait speed and for both 8-m walks with uniform test protocols in maximal and usual gait speed. While the overall distance of walks had no significant effect on usual gait speed, the test protocol as well as the interaction of test protocol and distance did so.

In general, our results are in line with other findings and emphasize the importance of standardized test protocols for supervised gait speed measurements in research and clinical practice. Sustakoski et al., and Wang et al., found clinically meaningful and statistically significant effects of test protocols in similar populations, but with different instrumentation [13, 14]. Warden et al. [15] showed differences for usual (mean difference 0.05 m/s) and maximum (mean difference 0.16 m/s) gait speed in younger participants. Furthermore, Wang et al. revealed that gait speeds with dynamic protocols were comparable over shorter (4-m) and longer (10-m) distances [14]. In the present study, these findings could not be replicated over 4-and 8-m.

Although all aforementioned studies examined slightly different protocols, they showed a significant effect of the inclusion or exclusion of acceleration and deceleration phases on gait speed measurements. Correspondingly, the comparison of our, seemingly identical, 4-m walk tests with mixed setup conditions (i.e., static-dynamic with dynamic-static) already yielded significant differences, suggesting a sizeable impact of even marginal test protocol variations. Therefore, the influence of test protocols on gait speed measurements should be considered in future inter-study comparisons and be weighted as potential exclusion criterion in meta-analysis. Alternatively, results must be adjusted. In general, our results indicate that short walk tests, e.g., 4-m, might be more vulnerable to a change in the protocol compared to a longer walk, e.g., 8-m. Due to the regulatory norms propagating the SPPB as a standard measure, it can be expected that many clinicians will adopt the 4-m walk from a standing position as their “gold” standard. This is also likely based on the limited space often available in outpatient settings and general practitioner offices. Consequently, the use of short walk assessments may have a dilutive effect in a meta-analysis.

It is noteworthy that usual gait speed ranged up to 2.01 m/s in our cohort of older persons, which could be indicative for the influence of an observer effect. An emerging discussion is the need and possibility to use supervised and non-supervised gait speed in real-world environments as a standard measure. This development is also pushed by the universal deployment of sensors in smartphones and other wearable sensor technology such as wristband sensing. Recent findings show that measurements of supervised usual gait speed in controlled conditions such as outpatient and inpatient clinics are significantly faster than in gait speed measurements in daily life [7]. It is likely that the setting, the observer and contextual circumstances have a major influence on “normal”, “usual” or “habitual” walking and gait speed in particular.

## Limitations

To keep the physical burden for participants within tolerable limits and to avoid fatigue effects, the gait analysis was restricted to only eight walks and to only the gait speed parameter. As a result, not every possible test protocol combination over 4-and 8-m was examined. Furthermore, every condition was only assessed once, increasing the variance of the gait speed measurements compared to the mean of multiple trials. Future research could investigate the effect on other kinematic parameters.

The inclusion criteria might have produced a selection effect toward a participation of mainly physical healthy older persons. Additionally, some participants could have distorted the results due to over-motivation. Therefore, our results cannot be considered as a representation of the general population in the age range of ≥ 70 years, affecting the generalizability of the study.

## Conclusion

Gait speed was faster, if the test protocol did not include a static start or stop and this difference was greater for a shorter walking distance. This aspect must be regarded for comparison of study results and for future meta-analyses.

## Data Availability

The datasets generated during this study are not publicly available, but are available from the corresponding author on reasonable request.
